# Recurrent metastatic malignant peripheral nerve sheath tumor associated with neurofibromatosis I: a rare case with atypical immunohistochemistry^؆^

**DOI:** 10.1016/j.abd.2025.501199

**Published:** 2025-08-22

**Authors:** Luis Regagnan Dias, Beatriz Zimermano Coimbra, Marcelo Tiezzi, Mayndra Mychelle Landgraf, Marilda Aparecida Milanez Morgado de Abreu

**Affiliations:** a Department of Dermatology, Hospital Regional de Presidente Prudente, Presidente Prudente, SP, Brazil; b Stricto Sensu Postgraduate Program in Health Sciences, Universidade do Oeste Paulista, Presidente Prudente, SP, Brazil; c Department of Pathology, Hospital Regional de Presidente Prudente, Presidente Prudente, SP, Brazil; d Department of Oncology, Hospital Regional de Presidente Prudente, Presidente Prudente, SP, Brazil

Dear Editor,

Malignant Peripheral Nerve Sheath Tumor (MPNST) is an extremely rare tumor (1/100,000) that represents up to 10% of sarcomas, divided into those related to Neurofibromatosis type 1 (NF1), which corresponds to 50% of cases, and sporadic ones.^1^, ^2^ The most widely accepted hypothesis today is that NF1-related MPNST arises from a neurofibroma that undergoes multiple genetic and phenotypic alterations until transforming into a malignant lesion. It presents diagnostic criteria that are not yet consolidated in the literature due to its variable morphological, immunohistochemical (IHC), and genetic pattern, which is not yet fully elucidated.^2^, ^3^ Thus, this case demonstrates an evolution of NF1-related MPNST, the diagnostic difficulties, as well as the role of the dermatologist in the final diagnosis of the neoplasm.

A 43-year-old white male patient, a waiter with no known comorbidities, complained of a progressively growing mass on his right thigh for three months, with intense local pain associated with the externalization of the tumor 30 days before (Fig. 1). Dermatological examination revealed a violaceous tumor mass measuring approximately 23 cm with areas of hemorrhage, necrosis, and a fistulous tract with fetid serosanguineous drainage.

**Figure 1:**
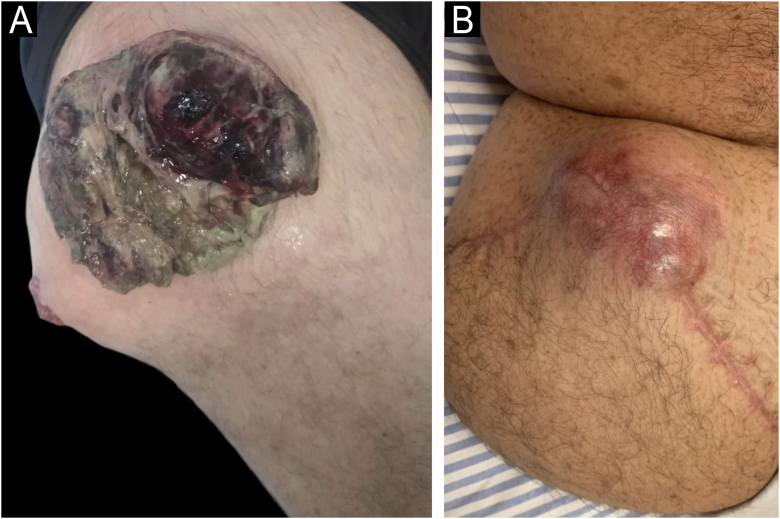
Tumor mass respectively: (A) on the day of admission and (B) recurrence 68 days after surgery.

Patient was hospitalized for diagnostic elucidation, chest and abdominal CT scans were performed, which were normal. MRI of the right thigh demonstrated a large, septate, heterogeneous expansile mass located in the region of the extensor muscles with peripheral contrast enhancement (Fig. 2).

**Figure 2:**
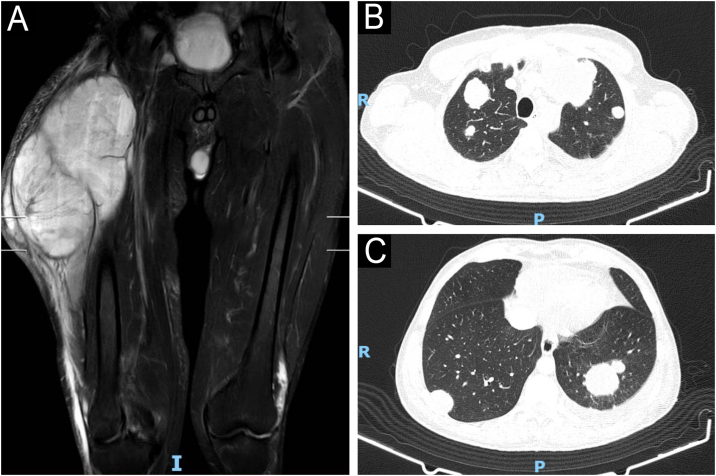
(A) Pre-surgery MRI of the thigh showing an expansile mass in the region of the extensor muscles measuring approximately 23.3 by 12.8 × 13.0 cm, with a volume of approximately 2000 mL, with a heterogeneous liquid content, areas suggestive of hemorrhage, and peripheral contrast uptake. (B and C) Contrast-enhanced chest computed tomography performed 68 days after complete excision of the lesion showed several pulmonary nodules with a neoplastic appearance.

Right rip disarticulation and inguinal lymphadenectomy were performed uneventfully. The pathology and IHC results (Fig. 3) were consistent with poorly differentiated sarcoma, with a spindle cell pattern, high cellularity, moderate atypia, fibrillar and edematous stroma, a mitotic index of 8 per 2 mm, diffusely positive CD34, diffusely positive monoclonal H3K27me3, Sox10-, S100-, Desmin-, and Myogenin-, suggesting a possible diagnosis of MPNST, especially if there are clinical signs of NF1-type genetic comorbidity.

**Figure 3:**
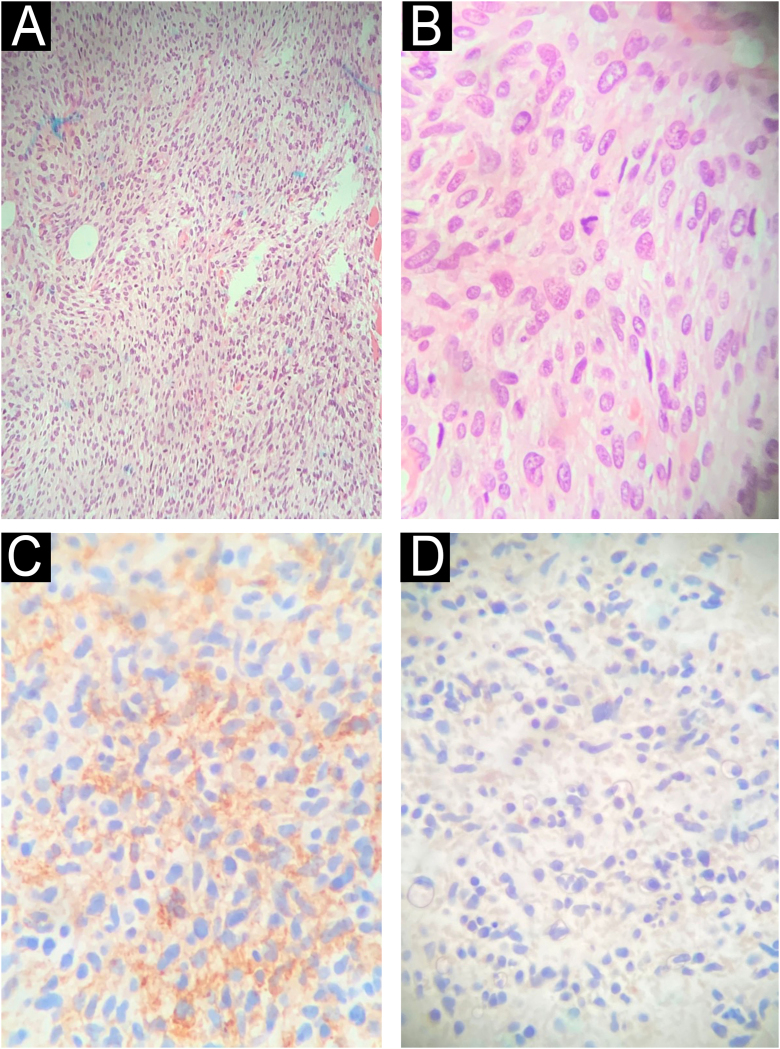
(A and B) Excisional fragment of a right hip disarticulation product stained with hematoxylin and eosin, ×100 and ×400, respectively, demonstrating proliferation of spindle cells with high cellularity, mitotic index of 8 per 2 mm^2^, moderate atypia, forming fascicles with variable, narrow elongations, in fibrillar and edematous stroma. (C) Immunohistochemistry of the specimen weakly positive for CD34. (D) Immunohistochemistry of the specimen negative for S100.

The patient returned with regrowth of the tumor lesion 68 days after surgery (Fig. 1). Upon re-admission, he was evaluated by the Dermatology team, and revealed multiple café-au-lait macules, multiple cutaneous neurofibromas, axillary freckles, and Lisch nodules in both irises. Diagnosis was confirmed by the National Institutes of Health criteria for NF1 (Fig. 4), which, together with the anatomopathological pattern and IHC, confirmed the diagnosis of MPNST.^3^ New staging exams were performed, which demonstrated recurrence of the local tumor associated with iliac lymph node involvement and pulmonary metastasis, recurrent stage IV T4 N1 M1 (Fig. 2). Palliative chemotherapy with ifosfamide and doxorubicin was initiated; the patient remains under multidisciplinary follow-up.

**Figure 4:**
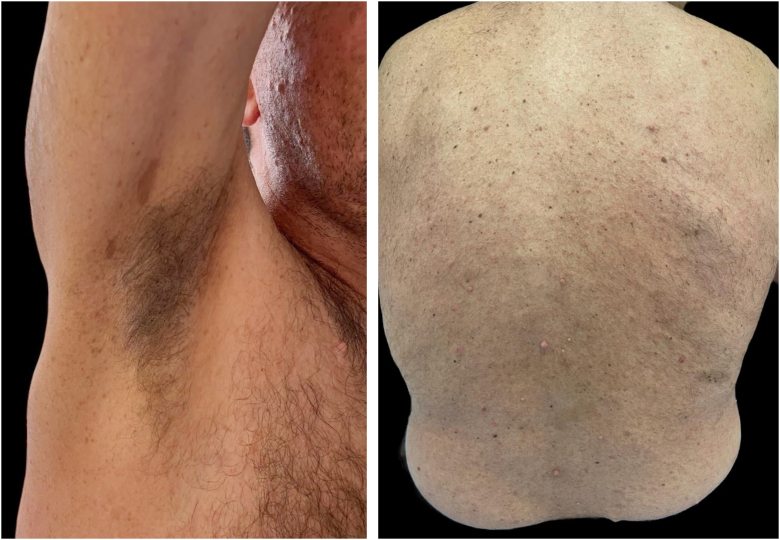
Presence of multiple superficial cutaneous neurofibromas, axillary freckles and multiple café-au-lait spots.

The diagnosis of MPNST remains a challenge, given its clinical behavior similar to that of other sarcomas. Anatomopathological examination is nonspecific and aims to exclude other tumor diagnoses, with heterogeneous microscopy findings of a high mitotic index, nuclear atypia, necrosis, and hemorrhage being common. On IHC, S-100 is positive in 50%–60% of cases; however, its negative expression indicates a higher mortality and risk of metastasis^4^, ^5^; SOX10 is present in 70% of cases; however, when the expression is negative, it also impacts a worse prognosis^6^; CD34 is weakly positive in high-grade sarcomas^7^; complete loss of H3K27me3 expression occurs in 30%–90%, with positivity being more common in those related to NF1.^8^ Negative expression of desmin and myogenin is important for the diagnostic exclusion of “Triton”-type MPNST and rhabdomyosarcoma.^9^

No defining markers for the disease have yet been discovered. The above case was a major challenge due to the late discovery of NF1, which was only made after tumor recurrence.

When compared with other reports in the literature, this case stands out on IHC, presenting an atypical set of findings, previously outside the patterns reported in MPNST related to NF1.^10^ Negative for S100 and SOX10, weakly positive CD34, and positive H3K27me3 are extremely rare and, as previously mentioned, indicative of a worse prognosis, which may explain the extremely aggressive behavior of the reported MPNST, which recurred with metastatic lesions 68 days after surgery. The survival rate for neurofibromatosis-related MPNST is estimated at 21% over the next five years,^2^ possibly lower in this metastatic patient with an atypical and even more aggressive IHC pattern.

The rarity of this case, as well as its diagnostic difficulty and severity, demonstrates the importance of such reports, so that the disease becomes more widely known among healthcare professionals, especially dermatologists who conduct ongoing follow-up of patients with NF1, those primarily affected by the tumor.

## Research data availability

Does not apply.

## Scientific associate editor

Hiram Larangeira de Almeida Jr.

## Financial support

None declared.

## Authors' contributions

Luis Regagnan Dias: Collection of data; analysis of data; critical review of the literature; drafting and editing of the manuscript.

Beatriz Zimermano Coimbra: Collection, analysis, and interpretation of data.

Marcelo Tiezzi: Critical review of the literature; intellectual participation in the introductory process.

Mayndra Mychelle Landgraf: Critical review of the literature; intellectual participation in the introductory process.

Marilda Aparecida Milanez Morgado de Abreu: Design and planning of the study; effective participation in research orientation; intellectual participation in the introductory process; approval of the final version of the manuscript.

## Conflicts of interest

None declared.
